# Plasma Lithium Levels in the General Population: A Cross-Sectional Analysis of Metabolic and Dietary Correlates

**DOI:** 10.3390/nu12082489

**Published:** 2020-08-18

**Authors:** Janna Enderle, Urte Klink, Romina di Giuseppe, Manja Koch, Ulrike Seidel, Katharina Weber, Marc Birringer, Ilka Ratjen, Gerald Rimbach, Wolfgang Lieb

**Affiliations:** 1 Institute of Epidemiology, Kiel University, 24105 Kiel, Germany; janna.enderle@epi.uni-kiel.de (J.E.); urteklink@posteo.de (U.K.); rominadigiuseppe@gmail.com (R.d.G.); mkoch@hsph.harvard.edu (M.K.); katharina.weber@epi.uni-kiel.de (K.W.); ilka.ratjen@epi.uni-kiel.de (I.R.); 2 Department of Nutrition, Harvard T.H. Chan School of Public Health, Boston, MA 02115, USA; 3 Institute of Human Nutrition and Food Science, Kiel University, 24118 Kiel, Germany; seidel@foodsci.uni-kiel.de (U.S.); rimbach@foodsci.uni-kiel.de (G.R.); 4 Department of Nutritional, Food and Consumer Science, University of Applied Sciences Fulda, 36037 Fulda, Germany; marc.birringer@oe.hs-fulda.de; 5 Biobank PopGen, University Hospital Schleswig-Holstein, Campus Kiel, 24105 Kiel, Germany

**Keywords:** plasma lithium levels, dietary correlates, metabolic risk factors, renal function, general population

## Abstract

Initial evidence suggests that lithium might affect life expectancy and the risk for different disease conditions, but most studies were conducted in patients on lithium medication. Little is known about the association of blood lithium levels within the physiological range with cardiometabolic risk factors and diet. We measured plasma lithium in a community-based sample from Northern Germany (samples taken between 2010 and 2012). All participants (aged 25–82 years) underwent standardized examinations and completed a semi-quantitative food frequency questionnaire. Of several variables tested, the estimated glomerular filtration rate (eGFR) was statistically significantly (inversely) associated with lithium levels, mainly in individuals with slightly impaired renal function (eGFR < 75 mL/min/1.73 m^2^). Besides, lithium levels were positively associated with age and alcohol intake. Using reduced rank regression, we identified a dietary pattern explaining 8.63% variation in plasma lithium levels. Higher lithium levels were associated with higher intakes of potatoes, leafy vegetables, root vegetables, fruits, tea, beer, wine and dietetic products and lower intakes of pasta, rice, pork, chocolate, sweets, soft drinks, other alcoholic beverages, sauces and snacks. Our observations suggest that plasma lithium levels are associated inversely with kidney function, particularly in individuals with slightly impaired renal function, and positively with age and alcohol intake. Lithium at physiological levels was moderately related to an exploratory dietary pattern.

## 1. Introduction

Lithium is an alkali metal in the Earth’s surface [1,2]. Due to its high reactivity, it does not naturally occur in elemental but usually in ionic form (e.g., as lithium chloride, lithium hydroxide or lithium carbonate) [1,3,4]. Lithium is present in trace amounts (7–200 µg/g) in soil and virtually all rocks [1]. Mobilized by rainfall and other weathering processes, it dissolves in groundwater, concentrating up to 500 µg/L, is taken up by plants and ultimately enters the food chain [1,5]. Lithium can be found in foods in concentrations that are both dependent on the type of food and geographical growing location [6,7,8].

The trace element lithium is absorbed in the intestinal tract and is typically present in all human organs and tissues, distributed in aqueous body fluids and is excreted by the kidneys [1,2]. A recent investigation into the bioavailability of lithium from mineral waters with different lithium content reported that the consumption of these waters resulted in a dose-dependent response in serum lithium concentrations and total urinary lithium excretion [9]. Whereas deficiency in lithium causes behavioral abnormalities and reduced litter size in rats [10], as well as reproduction and lactation problems in goats [11], human diseases associated with lithium deficiency are still unknown [12]. In contrast, a total lithium intake of up to 10 mg/day has shown no adverse effects on human health [4].

Nonetheless, the physiological functions of lithium and its essential role in humans and animals have not yet been fully established. A provisional RDA for a 70 kg adult of 1000 µg/day has been suggested [1]. However, as lithium has yet not been considered as an essential trace element, this recommendation is provisional and thus cannot be fully applied in dietary practice [2]. Epidemiological and experimental evidence suggests that lithium might exhibit a variety of health effects [2]. Dietary lithium may promote longevity in different model organisms such as *Drosophila melanogaster* [13]. Furthermore, exposure to low concentrations of lithium chloride increased the life span of the nematode *Caenorhabditis elegans* [12]. In humans, inverse associations of lithium levels in drinking water with all-cause mortality [12], the risk of dementia [14] and Alzheimer’s disease mortality [15] were reported. Lithium levels in drinking water have been correlated with lower suicide rates in several countries and regions around the world [5,16,17,18,19].

In modern pharmacotherapy, lithium is used to treat different psycho-neurological conditions, including bipolar disorders [2,20]. It has a relatively narrow therapeutic range and therapeutic lithium intake may be accompanied by adverse effects such as nausea, diarrhea, polyuria and polydipsia, tremor, weight gain, cognitive impairment and skin complaints [20,21]. Pharmacological lithium intake was found to be the leading cause of drug-induced nephrogenic diabetes, as well as associated with hypothyroidism and hyperparathyroidism [22,23,24,25]. However, the current evidence regarding the association of lithium intake with renal function is controversial. Several studies reported negative effects of long-term lithium treatment on kidney function [20,21,26,27,28,29]. However, in a recent meta-analysis, Paul et al. [30] found that any lithium-associated increase in serum creatinine, a biomarker used to estimate glomerular filtration rate (eGFR), is quantitatively small and of low clinical significance. By contrast, other studies reported no association between the risk of chronic kidney disease and lithium exposure or serum lithium levels [31,32]. However, the risk for chronic kidney disease through long-term lithium treatment has been reduced in recent years in parts by optimized treatment [33].

Serum lithium levels attributable to food and water intake are many times lower than those achieved through lithium treatment, and, as previously mentioned, may rather mediate preventive health effects. Many studies on the potential health effects of lithium use experimental designs utilizing cell cultures or animal studies, or are case reports. Thus far, only a few large scale studies with several hundred thousand individuals [12,14] and some studies with a few hundred participants [34,35,36] examined the role of lithium in humans. However, most of these studies focused on patients on lithium medication [21,24,26,28,29,33,37,38,39,40,41,42,43,44,45,46,47,48] rather than general population samples. Thus, there is little information on physiologic lithium concentrations and its relevance to physiological organ function or to disease processes in humans.

We, therefore, performed an exploratory analysis to investigate which dietary, anthropometric, metabolic and lifestyle factors correlate with circulating lithium concentrations (in the physiological range) in a moderate-sized community-based sample.

## 2. Materials and Methods

### 2.1. Study Sample

The analyses were conducted using data from the second examination cycle (conducted between 2010 and 2012) of the control sample (initially collected as a reference sample for genetic analyses) of the Biobank PopGen in Kiel, Northern Germany [49,50]. The initial sample (*n* = 1316; men and women between 18 and 80 years of age and recruited between 2005 and 2007) consisted of 747 individuals who were randomly selected from local population registries and of 569 blood-donors from the University Hospital in Kiel. Overall, 952 participants attended the second examination cycle and received comprehensive clinical and molecular phenotyping along with blood sampling, as detailed below. In addition, participants completed standardized questionnaires on demographics, lifestyle (including diet, education, physical activity and smoking status) and medical history.

Lithium plasma levels were available in 929 study participants. After the exclusion of one participant on lithium medication, the final sample consisted of 928 participants, which was used to study the association of lithium plasma levels with different sample characteristics. Another six participants were excluded from the dietary analyses due to missing dietary intake data, resulting in a sample size of 922 participants for these analyses.

The study was conducted in accordance with the Declaration of Helsinki and the Ethics Committee of the Medical Faculty of the University of Kiel approved all study procedures (Project identification code A 156/03). All participants gave written informed consent prior to their inclusion in the study.

### 2.2. Clinical Evaluation and Definitions

Trained personnel performed anthropometric measurements [50,51]. Bodyweight and height were measured with subjects wearing only light indoor clothing and no shoes. Body mass index (BMI) was calculated as weight (kilograms)/height (meters)^2^. Waist-to-hip-ratio was calculated by dividing waist circumference by hip circumference. Waist circumference was measured at the midpoint between the lower costal margin and the superior iliac crest [50].

Blood pressure was measured twice using a sphygmomanometer with the participants sitting for at least 5 min. Average systolic and diastolic blood pressures were calculated as the arithmetic mean. Hypertension was defined as systolic blood pressure ≥ 140 mmHg or diastolic blood pressure ≥ 90 mmHg or use of antihypertensive medication. Type 2 diabetes was defined as glycated hemoglobin (HbA_1c_) ≥ 6.5% (48 mmol/mol), or fasting serum glucose ≥ 126 mg/dL, or use of anti-diabetic medication.

Physical activity was classified in metabolic equivalent of task (MET)-hours per week by asking participants to report the weekly hours spent in walking, cycling, sports, gardening, home improvement activities and household tasks as well as stair climbing defined as floors per day [50], and then multiplied by the corresponding metabolic equivalent of task values and summed over all activities [52].

The Chronic Kidney Disease Epidemiology Collaboration (CKD-EPI) equation was used to estimate the eGFR [53].

### 2.3. Biochemical Measurements

Blood samples were taken from participants in a sitting position. HbA1c, plasma glucose, total, high-density lipoprotein (HDL) and low-density lipoprotein (LDL) cholesterol, triglycerides, C-reactive protein (CRP), Glutamate-Oxalacetate-Transaminase (GOT), Gamma-Glutamyl-Transferase (GGT) and Glutamate-Pyruvate-Transaminase (GPT) were measured on the same day in unfrozen blood samples at the Institute for Clinical Chemistry, University Hospital Schleswig-Holstein Campus Kiel [54].

A total of 352 out of the 928 participants had CRP values below the detection limit (0.9 mg/L). For these values, we assigned participants half of the detection limit as their CRP value.

For biomarker analyses in plasma, blood samples were drawn into plasma separator tubes (Sarstedt AG, Nümbrecht, Germany), centrifuged, aliquoted and stored at −80 °C until analyses. Lithium plasma levels were determined via an inductively coupled plasma-mass spectrometry (ICPMS) ICAP Q instrument (Thermo Fisher Scientific, Waltham, MA, USA) and conducted by SYNLAB (Jena, Germany). Measurements were conducted in accordance with DIN EN ISO 17294-2: 2017-01. Plasma samples were diluted 1 to 50 and stabilized with 2-propanol and Triton X-100. Rhodium (2 µg/L) was added as the internal standard. The limit of detection (LOD) of plasma samples was 0.1 µg/L. To increase the sensitivity, the use of the mass spectrometry collision chamber was neglected, and the LOD was lowered to 0.002 µg/L. The ICPMS lithium analysis was fully validated (Accuracy: 98.8%, SD = 0.7%, *n* = 6; Precision: 1%), within a ring trial of the German External Quality Assessment Scheme (G-EQUAS). Matrix certified reference material of such ring trials was used for specific method validation [55].

### 2.4. Dietary Assessment

A self-administered semi-quantitative 112 food and beverage items food frequency questionnaire (FFQ) was used to assess the dietary intake during the past 12 months. The FFQ was specifically designed and validated for German populations [50,56] and covered both intake frequencies and quantities of food consumption. The German Food Code and Nutrient Data Base (version II.3) was then used to assess nutrient and energy intake.

To simplify data interpretation and to minimize the within-person variations in individual food intakes, the 112 food items were assigned to 42 predefined food group categories according to the similarity of nutrient characteristics and/or culinary usage [50]. Data on the intake of the 42 food groups were available for 922 participants.

### 2.5. Statistical Analysis

Normally distributed variables were reported as mean and standard deviation (SD) or 95% confidence interval (CI); right-skewed variables were reported as the median and interquartile range (IQR); and categorical variables were presented as absolute numbers and percentages. Missing values were imputed using sex-specific median values. Comparisons of sample characteristics and dietary intake across lithium tertiles were conducted using chi-square test, Kruskal–Wallis test or general linear model, as appropriate. Prior to statistical analysis, lithium plasma levels were log-transformed (natural logarithm) due to their skewed distribution.

To assess seasonal variations in lithium plasma levels, we created categories corresponding to the four seasons based on the month the study participants underwent examinations (winter, January–March; spring, April–June; summer, July–September; and fall, October–December).

To select a parsimonious model with the highest predictive power of circulating plasma lithium levels, we applied the Least Angle Regression (LAR) algorithm introduced by Efron and colleagues [57]. Briefly, the LAR algorithm starts by finding the predictor with the highest correlation with the response variable (log lithium). Successively, it moves the selected predictor towards its least square estimate, until the algorithm finds another predictor equally correlated with the model residual. It then adds this predictor to the model, and the least square estimate starts again with both variables. When all the variables a priori included in the regression model are tested, the LAR algorithm stops, and the predictors whose coefficients are shrunk to zero are removed from the set and the process starts again [57].

We included the following variables in the regression model: age, sex, waist circumference regressed on BMI, systolic and diastolic blood pressure, total cholesterol, HDL cholesterol, triglycerides, HbA1c, eGFR, CRP, GOT, GGT, GPT, physical activity, education, smoking status and daily alcohol intake.

We used the restricted cubic spline regression (RCS) functions to investigate potential associations of LAR-selected correlates with circulating plasma lithium concentrations. Four knots were used, located at the 5th, 35th, 65th and 95th percentiles. We included the variables in the model selected by LAR algorithm as those explain most of the variation in circulating plasma lithium levels. Of these, categorical variables such as sex and smoking status were included as adjusting variables in the final model, while other predictors were modeled according to the spline functions. The RCS regression models fitted with generalized estimating equations were constructed using the %RCS_Reg SAS macro developed by Desquilbet and Mariotti [58].

All analyses were performed with SAS^®^ Enterprise Guide 7.1 (SAS Institute, Cary, NC, USA).

### 2.6. Exploratory Dietary Pattern

Reduced rank regression (RRR) was used to identify a dietary pattern explaining the variation in circulating lithium levels. RRR applied in nutritional epidemiology aims to identify factors from food intake that explain the maximum variation in pre-specified intermediate response variables. RRR can be applied to explore potential biological mechanisms for the diet-disease associations due to its ability to incorporate the intermediate response variables. As described by Hoffmann and colleagues [59], RRR extracts linear functions of predictors that explain as much response variation as possible, and it produces as many patterns as there are response variables. In the present study, the RRR was implemented to derive a dietary pattern from the 42 above-mentioned food groups (predictor variables) with plasma lithium levels as response variable. The correlations between the extracted factor and foods are called factor loadings, and food groups with an absolute factor loading of |0.15| were considered part of the dietary pattern explaining the variation in circulating lithium levels.

## 3. Results

### 3.1. General Characteristics

Anthropometric and metabolic measures as well as lifestyle characteristics of the study sample are shown in Table 1. The mean age of the population was 61 (SD 13) years and the sample included slightly more men than women (*n* = 532, 57.3%). The median plasma concentration of lithium was 0.96 (IQR 0.70–1.37) µg/L for the overall population and 0.92 (IQR 0.69–1.32) µg/L and 1.03 (IQR 0.72–1.43) µg/L for men and women, respectively.

### 3.2. Correlates of Circulating Lithium Concentrations

General characteristics according to lithium tertiles are presented in Table 2. We observed statistically significant trends of older age, higher HbA1c and a higher prevalence of diabetes, lower LDL cholesterol, lower eGFR and higher creatinine levels with increasing lithium tertiles. No seasonal variations in circulating plasma lithium levels were observed (Table S1). We further explored the association between lithium plasma levels and LDL levels. RCS analyses, adjusted for age and sex, provided no statistically significant evidence for an overall (*p* = 0.085) association or for a non-linear association (*p* = 0.41) of lithium concentration and LDL levels. Likewise, in a linear regression model, adjusting for age and sex, the association between lithium (exposure) and LDL cholesterol (outcome) was not statistically significant (β: −0.003; 95% CI: −0.01; 0.01; *p* = 0.48).

Subsequently, the LAR algorithm selected a parsimonious model of five predictors for plasma lithium concentration, namely age, eGFR, diastolic blood pressure, alcohol intake and smoking status, which explained 7.25% of the variation in circulating plasma lithium levels (Table 3).

**Figure 1:**
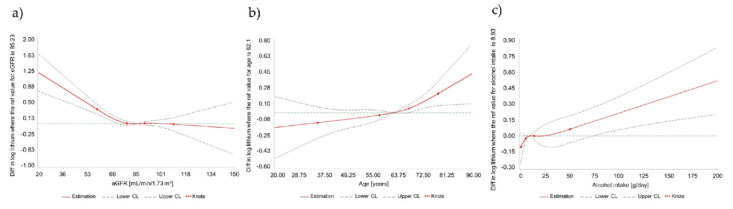
Association of log-transformed lithium plasma levels with eGFR, age and alcohol intake (each variable modeled individually) using RCS. *Legend to the Figure:* Restricted cubic splines (RCS) regression for the association of plasma lithium concentrations in 928 participants with: (**a**) estimated glomerular filtration rate (eGFR); (**b**) age; and (**c**) alcohol intake. The solid line indicates estimated differences in log lithium and dashed lines indicate 95% CI derived from RCS regression, with four knots placed at the 5th, 35th, 65th and 95th percentiles of the distribution, using the 50th percentile as a reference, of: (**a**) eGFR values; (**b**) age; and (**c**) alcohol intake. Estimated differences were adjusted for age, sex, diastolic blood pressure, eGFR, alcohol intake and smoking status. Wald *p* values are: (**a**) eGFR, *p* for nonlinearity 0.0006 and *p* for overall association <0.0001; (**b**) age, *p* for nonlinearity 0.29 and *p* for overall association 0.002; and (**c**) alcohol intake, *p* for nonlinearity 0.46 and *p* for overall association 0.002.

RCS functions were then used to model the relations of these predictors with circulating lithium concentrations. In this regression model, eGFR remained statistically significantly associated with circulating lithium levels in a non-linear fashion (*p* = 0.0006) (Figure 1a). Below a threshold of about 75 mL/min/1.73 m^2^, eGFR displayed an inverse association with circulating lithium levels. Above this threshold, no such association was observed. Furthermore, we observed statistically significantly higher lithium concentrations (median (Q1; Q3): 1.43 (1.03, 2.02) µg/L) in individuals with eGFR below 60 mL/min/1.73 m^2^ (*n* = 57) as compared to individuals with eGFR ≥ 60 mL/min/1.73 m^2^ (median: 0.93 (0.69, 1.33) µg/L; *p* < 0.0001), in unadjusted analyses.

RCS functions further revealed an overall significant association of age and alcohol intake with circulating lithium levels, respectively (Figure 1b,c).

### 3.3. Dietary Correlates of Circulating Lithium Concentrations

Table 4 shows the intake of 42 food groups according to lithium tertiles. Participants in the third lithium tertile consumed more root vegetables, beer, wine and tea than those in the first and second tertile. Besides, for fish and fish products, eggs and sugar products, we observed significantly different intakes across tertiles of plasma lithium.

We additionally performed RRR to identify a dietary pattern from the intake of 42 food groups, which explains the variation in circulating plasma lithium levels (Table 5). Using lithium levels as response variable, we derived a dietary pattern characterized by high intakes of potatoes, leafy vegetables, root vegetables, fruits, tea, beer, wine and dietetic products and low intakes of pasta and rice, pork, chocolate and sweets, soft drinks, other alcoholic beverages, sauces and snacks. The dietary pattern explained 8.63% of the variation in circulating lithium levels.

## 4. Discussion

We assessed dietary, anthropometric, metabolic and lifestyle correlates of circulating lithium levels in a moderate sized community-based sample (*n* = 928) from the community.

### 4.1. Principal Observations

Our main observations were as follows: First, we observed statistically significant and directionality-consistent trends across lithium tertiles for the following variables: mean values for age, HbA1c, creatinine and the prevalence of diabetes rose, while mean LDL cholesterol and eGFR decreased with increasing lithium tertiles. Second, multivariable-adjusted RCS regressions revealed an inverse association of lithium levels with eGFR and positive associations with age and alcohol intake. Third, using lithium levels as response variable, we identified a dietary pattern characterized by higher intakes of potatoes, leafy vegetables, root vegetables, fruits, tea, beer, wine and dietetic products and lower intakes of pasta and rice, pork, chocolate and sweets, soft drinks, other alcoholic beverages, sauces and snacks as being associated with higher lithium levels.

### 4.2. In the Context of the Published Literature

Our observation of an inverse association of plasma lithium levels with eGFR agrees with analyses conducted in defined patient groups. Many studies included patients taking lithium medications, and some investigations reported an association between lithium medication and a reduced eGFR, along with an increased risk of developing chronic kidney disease [26,27,29,45]. However, these patients were treated with high lithium dosages, resulting in circulating plasma levels which were roughly 1000 times higher than physiological lithium concentrations (as, e.g., in our sample).

An important feature of the present analysis is, therefore, that we assessed the correlation of lithium concentrations within the physiological range in a community-based sample. In addition, in agreement with our observations, a recent investigation by Seidel et al., reported a dose–response relation between the amount of lithium ingested through mineral waters and the amount excreted by the kidneys. Initial lithium plasma levels of their study participants were in the same range as our participants [9].

Furthermore, in the present analysis, participants with higher circulating lithium levels not only had slightly lower eGFR but were also found to be older. As kidney function declines with age [60] and since circulating lithium is excreted by the kidneys, it is conceivable that slightly more lithium is retained in the plasma of elderly people. These findings were supported by Kapusta and colleagues, who showed that lithium excretion depends on the eGFR and can, therefore, be reduced with age and in individuals with renal disease [5]. In the kidneys, lithium is filtered from the glomerular capillaries and is mainly reabsorbed in the proximal tubule [61]. Thus, an impaired eGFR might inhibit the glomerular filtration of lithium and increases lithium retention in the blood.

### 4.3. Association of Lithium Levels with Anthropometric, Metabolic and Lifestyle Characteristics

To our knowledge, there is no epidemiological study investigating the association between lithium plasma levels (within the physiological range) and various anthropometric, metabolic and lifestyle characteristics in the general population. Experiments in model organisms have shown that lithium exposure increased their life span, while, in a Japanese population, an inverse relationship between lithium levels in drinking water and all-cause mortality has been reported [12,13]. The underlying mechanisms for these associations are largely unknown and deserve further investigations. Therefore, we assessed the association of a broad spectrum of potential correlates with plasma lithium concentrations.

We observed some differences in anthropometric, metabolic and lifestyle characteristics according to tertiles of circulating lithium. However, in multivariable-adjusted RCS regressions including the LAR-selected correlates, eGFR and age remained statistically significantly associated with plasma lithium levels. Specifically, we were able to demonstrate that, for individuals with an eGFR below ~75 mL/min/1.73 m^2^, i.e., mildly decreased eGFR (Stage 2) [62], circulating plasma lithium levels were inversely associated with eGFR. However, such an association between eGFR and lithium levels was not evident for eGFR values above ~75 mL/min/1.73 m^2^. Thus, a slight impairment in renal function, as, e.g., observed with aging [60], might lead to less lithium excretion and a tendency towards higher lithium plasma levels. In line with this concept, we observed higher lithium plasma levels in participants with Stage 3 chronic kidney disease (eGFR < 60 mL/min/1.73 m^2^), as compared to individuals with eGFR ≥ 60 mL/min/1.73 m^2^.

As some investigations reported seasonal differences in circulating lithium levels in psychiatric patients on lithium medication [63,64], we also looked at seasonal variation in our sample. However, no seasonal variation in plasma lithium levels was observed.

### 4.4. Dietary Correlates of Plasma Lithium Concentrations

Since many healthy foods have been suggested as sources of lithium [2], we also performed a RRR to derive a dietary pattern explaining the variation in lithium levels. The purpose of this analysis was to understand which food combination explains the highest variation in plasma lithium. For the overall sample, the derived dietary pattern was characterized by positive loadings for potatoes, leafy vegetables, root vegetables, fruits, tea, beer, wine and dietetic products and negative loadings for pasta and rice, pork, chocolate and sweets, soft drinks, other alcoholic beverages, sauces and snacks. Interestingly, we observed higher plasma lithium concentrations with higher tea consumption, although tea contains rather low amounts of lithium [55]. Tea, e.g., green tea, is known to exhibit, e.g., antioxidant, anti-inflammatory or metal chelating properties, and isolated compounds have convincingly been shown to beneficially affect kidney function in humans [65]. Thus, possible beneficial effects of dietary tea consumption on renal function are also conceivable, but warrant confirmation in large human trials [66,67,68,69]. The dietary pattern we derived explained almost 9% of the inter-individual variation in plasma lithium levels. Data by Cai and coworkers suggest that a diet rich in proteins, fruits, vegetables and beer may partly counteract a decline in eGFR in the general population which is partly in line with the present observations [70].

To our knowledge, this is the first epidemiologic study that identified a dietary pattern explaining the variation in plasma lithium levels. Some studies have investigated the lithium content of different foods but have reported varying results. A recent analysis of different beverages revealed that, compared to different mineral waters, beverages such as soft drinks, beer, wine and hot beverages have many times lower lithium concentrations [55]. The highest concentration of lithium has been found in nuts, pastries as well as cold meat and sausages [8], but also in samples of tofu and fish [7]. Grains, vegetables, dairy products and meat have also been considered major dietary sources. Particularly vegetables grown directly in the soil may contain more lithium than foods that are otherwise produced [1]. Lithium content in the soil is highly dependent on the geographical location, which ultimately affects the lithium content in foods [2]. However, lithium content in soil and water in Northern Germany and around Kiel is low [9], and in the present study it is not known where the foods the participants consumed were grown or produced. Robust lithium food databases are needed to better evaluate dietary lithium intake in humans in future studies.

### 4.5. Strengths and Limitations

Strengths of our study include the moderate-sized community-based sample (most prior studies were conducted in selected patient groups on lithium medication) and the comprehensive characterization of our study participants with respect to diet, anthropometry and metabolic traits. With regard to the statistical methods used, we implemented the LAR algorithm as a variable selection method, which has been shown to be superior compared to other stage-wise regression techniques [57]. Through RCS analysis, we were able to model linear and non-linear relationships of the selected predictors, mutually adjusted for each other, with circulating lithium levels.

However, the following limitations merit consideration. Although we can raise hypotheses for further investigations, the cross-sectional nature of the present study does not allow causal inferences. Plasma lithium levels and the FFQ, used in the present study to derive the dietary pattern, refer to different time intervals, i.e., the FFQ inquires the food intake in the past year while the half-life of plasma lithium after dietary intake is below 24 h [9]. Several factors have been proposed to influence circulating lithium levels, including dietary intake of sodium xanthines (theophylline and caffeine) and various medications [2]. In our study, we were unable to investigate potential relations of these factors with lithium levels as these measures were not available. Besides, other unmeasured biomarkers and/or other unknown confounding variables might have influenced circulating plasma lithium levels. Furthermore, our sample comprises adults from Northern Germany, thus limiting the generalizability of the present findings to other populations and/or age groups. In this context, we have initial evidence that the lithium concentration in the local drinking water is rather low as compared to other regions in the world [9,35]. If lithium concentrations in drinking water should be decisive for circulating lithium concentration, the rather low concentrations in the local drinking water might explain why we observed an association of many other dietary factors with plasma lithium concentrations. Due to lack of robust database with lithium concentration and lack of information on the origin of foods, we did not calculate dietary lithium intake. However, as lithium is directly taken up from the soil, especially plant foods are rich in lithium [1], and the dietary pattern we identified as being associated with plasma lithium concentration is characterized by a rather high intake of plant derived foods.

## 5. Conclusions

In a general population sample from Northern Germany, we observed that higher plasma lithium levels were associated with higher age and a lower eGFR, particularly in individuals with slightly impaired renal function (eGFR < 75 mL/min/1.73 m^2^). Furthermore, the derived dietary pattern explaining the variation in circulating plasma lithium levels was characterized by high factor loadings for healthy food groups, for example leafy and root vegetables, fruits and tea. While these findings suggest that lithium, at physiological plasma levels, might not be related to selected metabolic risk factors (upon multivariable adjustment), it is moderately related to an exploratory dietary pattern.

## Figures and Tables

**Table 1:** General characteristics of the overall study sample and stratified by men and women.

	Overall	Men	Women
*n* ^a^	928	532 (57.3)	396 (42.7)
Plasma concentration of lithium (µg/L) ^b^	0.96 (0.70, 1.37)	0.92 (0.69, 1.32)	1.03 (0.72, 1.43)
Age (years) ^c^	61 ± 13	61 ± 12	60 ± 14
Body mass index (kg/m^2^) ^c^	27.5 ± 4.9	27.7 ± 4.0	27.2 ± 5.9
Waist circumference (cm) ^b^	96.1 (88.1, 105.0)	99.9 (93.2, 108.2)	89.8 (80.1, 99.3)
Waist-to-hip ratio ^b^	0.94 (0.88, 1.00)	0.99 (0.94, 1.03)	0.88 (0.83, 0.92)
Systolic blood pressure (mmHg) ^c^	140 ± 18	142 ± 18	137 ± 18
Diastolic blood pressure (mmHg) ^c^	85 ± 9	86 ± 9	84 ± 9
Prevalent hypertension ^a^	585 (63.0)	355 (66.7)	230 (58.1)
HbA1c (%) ^c^	5.73 ± 0.61	5.76 ± 0.67	5.70 ± 0.52
Glucose (mg/dL) ^b^	98.0 (91.0, 105.0)	99.0 (93.0, 107.0)	95.0 (89.0, 103.0)
Prevalent diabetes ^a^	78 (8.4)	54 (10.0)	24 (6.1)
C-reactive protein (mg/L) ^b^	1.30 (0.45, 2.60)	1.20 (0.45, 2.20)	1.40 (0.45, 3.10)
Triglycerides (mg/dL) ^b^	106.0 (77.0, 140.5)	111.0 (80.5, 148.0)	99.5 (72.0, 131.5)
Total cholesterol (mg/dL) ^c^	222.8 ± 41.1	217.1 ± 40.3	230.6 ± 40.9
High-density-lipoprotein cholesterol (mg/dL) ^c^	64.8 ± 18.1	58.0 ± 14.3	74.0 ± 18.5
Low-density-lipoprotein cholesterol (mg/dL) ^c^	131.4 ± 33.5	131.2 ± 33.3	131.5 ± 33.8
Estimated glomerular filtration rate (mL/min/1.73 m^2^) ^c^	84.4 ± 15.4	84.2 ± 15.1	84.7 ± 15.6
Creatinine (mg/dL) ^c^	0.88 ± 0.18	0.97 ± 0.17	0.76 ± 0.12
Current smokers ^a^	129 (13.9)	76 (14.3)	53 (13.4)
Physical activity (MET-hours/week) ^b^	89.2 (58.2, 129.0)	83.8 (55.0, 124.5)	96.3 (65.3, 137.6)
High education (≥ 11 years) ^a^	299 (32.2)	199 (37.4)	100 (25.3)
Alcohol intake (g/day) ^b^	8.9 (3.2, 18.4)	12.3 (5.5, 23.1)	5.4 (1.9, 12.3)
Glutamate-Oxalacetate-Transaminase (U/L) ^b^	26.0 (22.0, 30.0)	27.0 (23.0, 32.0)	24.0 (21.0, 29.0)
Gamma-Glutamyl-Transferase (U/L) ^b^	25.0 (18.0, 37.0)	28.0 (21.0, 42.0)	19.0 (14.0, 29.5)
Glutamate-Pyruvate-Transaminase (U/L) ^b^	23.0 (17.0, 31.0)	26.0 (20.0, 34.0)	19.0 (15.0, 25.0)

Values are ^a^ *n* (%), ^b^ median (Q1; Q3) or ^c^ mean ± standard deviation. Abbreviations: HbA1C, glycated hemoglobin; MET, metabolic equivalent of task; Q, quartile.

**Table 2:** General characteristics of the overall study sample according to tertiles of circulating plasma lithium concentrations (*n* = 928).

Plasma Concentration of Lithium (µg/L)		T1 ≤ 0.78 µg/L	T2 0.79–1.21 µg/L	T3 ≥ 1.22 µg/L	*p* *
*n* (% male) ^a^		309 (61.2)	309 (57.9)	310 (52.9)	0.11
Plasma concentration of lithium (µg/L) ^b^		0.63 (0.52, 0.70)	0.95 (0.87, 1.08)	1.60 (1.37, 2.10)	<0.0001
Age (years) ^c^		57 ± 12	62 ± 12	64 ± 12	<0.0001
Body mass index (kg/m^2^) ^c^		28.2 ± 5.4	26.9 ± 4.4	27.3 ± 4.8	0.004
Waist circumference (cm) ^b^	male	100.3 (93.3, 108.8)	99.1 (92.7, 106.5)	100.2 (94.1, 108.5)	0.58
	female	92.0 (80.4, 101.0)	88.4 (79.0, 97.4)	89.3 (82.5, 98.8)	0.19
Waist-to-hip ratio ^b^	male	0.99 (0.95, 1.02)	0.99 (0.94, 1.03)	0.99 (0.94, 1.03)	0.94
	female	0.88 (0.83, 0.93)	0.87 (0.81, 0.91)	0.88 (0.83, 0.93)	0.20
Systolic blood pressure (mmHg) ^c^		141 ± 18	139 ± 19	140 ± 19	0.58
Diastolic blood pressure (mmHg) ^c^		86 ± 9	84 ± 9	85 ± 9	0.07
Prevalent hypertension ^a^		198 (64.1)	178 (57.6)	209 (67.4)	0.04
HbA1c (%) ^c^		5.65 ± 0.60	5.73 ± 0.54	5.82 ± 0.67	0.002
Glucose (mg/dL) ^b^		96.0 (90.0, 105.0)	98.0 (91.0, 105.0)	98.0 (92.0, 105.0)	0.25
Prevalent diabetes ^a^		17 (5.5)	23 (7.4)	38 (12.3)	0.01
C-reactive protein (mg/L) ^b^		1.40 (0.45, 2.90)	1.10 (0.45, 2.50)	1.30 (0.45, 2.40)	0.16
Triglycerides (mg/dL) ^b^		103.0 (77.0, 137.0)	106.0 (73.0, 140.0)	107.0 (80.0, 145.0)	0.47
Total cholesterol (mg/dL) ^c^		223.5 ± 41.4	223.8 ± 38.9	221.2 ± 43.0	0.71
High-density-lipoprotein cholesterol (mg/dL) ^c^		62.7 ± 16.8	66.6 ± 18.6	65.1 ± 18.6	0.03
Low-density-lipoprotein cholesterol (mg/dL) ^c^		134.8 ± 33.2	131.4 ± 31.7	127.9 ± 35.1	0.04
Estimated glomerular filtration rate (mL/min/1.73 m^2^) ^c^		89.0 ± 14.3	84.1 ± 14.1	80.2 ± 16.4	<0.0001
Creatinine (mg/dL) ^c^		0.87 ± 0.17	0.88 ± 0.16	0.91 ± 0.21	0.02
Current smokers ^a^		45 (14.6)	47 (15.2)	37 (11.9)	0.46
Physical activity (MET-hours/week) ^b^		86.5 (54.8, 123.0)	94.5 (63.3, 132.6)	85.4 (55.1, 134.3)	0.07
High education (≥11 years) ^a^		96 (31.1)	112 (36.3)	91 (29.4)	0.33
Alcohol intake (g/day) ^b^		8.6 (2.8, 15.8)	9.3 (3.8, 18.8)	8.8 (3.2, 20.7)	0.17
Glutamate-Oxalacetate-Transaminase (U/L) ^b^		26.0 (22.0, 30.0)	26.0 (22.0, 30.0)	26.0 (23.0, 31.0)	0.21
Gamma-Glutamyl-Transferase (U/L) ^b^		25.0 (17.0, 35.0)	23.0 (17.0, 35.0)	26.0 (18.0, 44.0)	0.07
Glutamate-Pyruvate-Transaminase (U/L) ^b^		24.0 (18.0, 32.0)	21.0 (17.0, 29.0)	22.0 (17.0, 30.0)	0.12

Values are ^a^ *n* (%), ^b^ median (Q1; Q3), ^c^ mean (95% CI). * *p*-values based on chi-square test, Kruskal–Wallis test (skewed variables) or general linear models (normally distributed variables). Abbreviations: CI, confidence interval; HbA1c, glycated hemoglobin; MET, metabolic equivalent of task; Q, quartile; T, tertile.

**Table 3:** Selected predictors for lithium plasma concentration by using Least Angle Regression algorithm.

Least Angle Regression	
R^2^	0.0725
Characteristic	Difference in plasma lithium concentration (µg/L) per 1-unit increment in the characteristic
Age (years)	0.0028
Diastolic blood pressure (mmHg)	−0.0016
Estimated glomerular filtration rate (mL/min/1.73 m^2^)	−0.0069
Smoking status (former vs. never smoking)	0.0140
Alcohol intake per drink (14 g/day)	0.0184

**Table 4:** Age- and sex-adjusted intake of 42 food groups [g/day] according to tertiles of plasma lithium concentrations (*n* = 922).

	Plasma Concentration of Lithium [µg/L]			
Food Groups [g/day]	T1 ≤ 0.78 µg/L *n* = 307	T2 0.79–1.21 µg/L *n* = 308	T3 ≥ 1.22 µg/L *n* = 307	*p* *
Potatoes	83.2 (78.7, 87.8)	84.4 (80.0, 88.9)	81.0 (76.5, 85.5)	0.56
Leafy vegetables	14.4 (12.7, 14.9)	16.1 (16.6, 14.9)	16.4 (14.7, 18.1)	0.14
Fruiting vegetables	92.6 (86.2, 99.0)	94.7 (88.4, 101.0)	93.3 (87.0, 99.6)	0.90
Root vegetables	22.1 (20.4, 23.7)	23.4 (21.8, 25.0)	25.4 (23.8, 27.1)	0.02
Cabbage	25.7 (24.1, 27.4)	27.4 (25.8, 29.1)	26.0 (24.4, 27.6)	0.29
Other vegetables	40.3 (38.1, 42.4)	39.7 (37.6, 41.8)	40.2 (38.1, 42.3)	0.93
Legumes	3.0 (2.6, 3.4)	3.1 (2.7, 3.4)	3.1 (2.8, 3.5)	0.89
Fruits	216.1 (198.4, 233.8)	232.9 (215.6, 250.3)	229.9 (212.4, 247.4)	0.37
Nuts and seeds	3.8 (3.2, 4.4)	4.3 (3.7, 4.9)	3.9 (3.3, 4.5)	0.51
Milk	110.6 (95.9, 125.3)	108.9 (94.5, 123.3)	104.5 (89.9, 119.0)	0.84
Dairy products	105.1 (97.1, 113.0)	110.6 (102.8, 118.3)	105.0 (97.1, 112.8)	0.52
Cheese	34.7 (32.6, 36.7)	37.9 (35.9, 40.0)	35.3 (33.2, 37.3)	0.06
Bread	116.4 (107.0, 125.9)	118.6 (109.3, 127.8)	111.2 (101.8, 120.5)	0.53
Pasta and rice	35.9 (33.0, 38.8)	37.2 (34.3, 40.1)	34.4 (31.5, 37.3)	0.40
Other cereals	12.6 (11.6, 13.5)	13.4 (12.5, 14.4)	12.6 (11.6, 13.5)	0.34
Beef	16.4 (14.5, 18.2)	18.5 (16.7, 20.4)	17.6 (15.7, 19.4)	0.28
Pork	34.4 (30.4, 38.3)	33.8 (30.0, 37.7)	32.9 (28.9, 36.8)	0.87
Poultry	14.6 (13.2, 15.9)	13.6 (12.2, 14.9)	14.4 (13.1, 15.8)	0.54
Processed meat	50.5 (46.9, 54.0)	49.3 (45.8, 52.8)	49.7 (46.2, 53.2)	0.89
Other meat	4.5 (4.0, 4.9)	4.8 (4.3, 5.2)	4.4 (4.0, 4.8)	0.44
Fish, fish products	25.8 (23.4, 28.3)	30.4 (28.0, 32.9)	29.5 (27.0, 31.9)	0.03
Eggs	18.3 (17.0, 19.7)	16.0 (14.7, 17.4)	18.0 (16.7, 19.4)	0.04
Butter	12.3 (10.8, 13.8)	12.4 (10.9, 13.9)	10.4 (8.9, 11.9)	0.11
Margarine	13.6 (12.0, 15.3)	13.9 (12.3, 15.5)	13.5 (11.9, 15.2)	0.94
Vegetable oils	8.6 (7.9, 9.3)	9.4 (8.7, 10.1)	9.2 (8.5, 9.9)	0.25
Other fats	2.2 (2.0, 2.3)	2.0 (1.9, 2.1)	2.0 (1.9, 2.1)	0.20
Sugar products (e.g., syrups, candy, ice cream, desserts)	40.2 (37.6, 42.8)	41.9 (39.3, 44.5)	37.1 (34.5, 39.7)	0.03
Chocolate sweets	12.5 (11.5, 13.5)	12.0 (11.0, 13.0)	11.1 (10.0, 12.1)	0.14
Cake, cookies	61.3 (56.5, 66.1)	61.6 (56.9, 66.3)	57.6 (52.9, 62.3)	0.43
Non-alcoholic beverages	1112.8 (1033.9, 1191.8)	1114.4 (1037.0, 1191.8)	1096.8 (1018.6, 1175.0)	0.94
Soft drinks	208.3 (137.4, 279.2)	190.1 (120.5, 259.7)	191.1 (120.8, 261.3)	0.92
Coffee	494.4 (450.4, 538.5)	488.5 (445.3, 531.7)	485.4 (441.8, 529.1)	0.96
Tea	192.1 (150.9, 233.3)	259.3 (218.9, 299.8)	313.9 (273.1, 354.7)	0.0003
Beer	116.2 (72.4, 160.0)	132.7 (89.7, 175.7)	212.7 (169.2, 256.1)	0.01
Wine	62.8 (41.7, 83.9)	90.3 (69.6, 111.0)	101.8 (80.9, 122.6)	0.03
Alcoholic beverages	17.1 (12.1, 22.0)	15.8 (11.0, 21.0)	11.4 (6.5, 16.3)	0.24
Sauces	51.9 (49.0, 54.9)	52.4 (49.4, 55.3)	49.2 (46.3, 52.1)	0.27
Soups	23.6 (21.4, 25.8)	24.3 (22.1, 26.5)	25.9 (23.8, 28.1)	0.32
Bouillon	17.6 (16.3, 18.9)	18.1 (16.8, 19.3)	18.7 (17.5, 20.0)	0.48
Soya products	2.1 (1.9, 2.4)	2.2 (2.0, 2.5)	2.4 (2.2, 2.7)	0.23
Dietetic products	0.5 (0.3, 0.6)	0.6 (0.4, 0.7)	0.7 (0.6, 0.9)	0.09
Snacks	1.0 (0.9, 1.1)	0.9 (0.8, 1.0)	0.9 (0.8, 1.0)	0.23

Data are expressed as Mean (95% confidence interval). * *p*-values are adjusted for age and sex and based on general linear models. Abbreviations: CI, confidence interval; T, tertile.

**Table 5:** Factor loadings in 42 food groups and explained variation in log-transformed lithium plasma concentration by using reduced rank regression (*n* = 922).

	Reduced Rank Regression Factor Loading
	Overall
Explained variation (%)	8.63
Food group (g/day)	
Potatoes	0.2169
Leafy vegetables	0.1641
Fruiting vegetables	-
Root vegetables	0.2354
Cabbage	-
Other vegetables	-
Legumes	-
Fruits	0.1894
Nuts and seeds	-
Milk	-
Dairy products	-
Cheese	-
Bread	-
Pasta and rice	−0.2083
Other cereals	-
Beef	-
Pork	−0.1670
Poultry	-
Processed meat	-
Other meat	-
Fish, fish products	-
Eggs	-
Butter	-
Margarine	-
Vegetable oils	-
Other fats	-
Sugar products (e.g., syrups, candy, ice cream, desserts)	-
Chocolate, sweets	−0.3668
Cake, cookies	-
Non-alcoholic beverages	-
Soft drinks	−0.2014
Coffee	-
Tea	0.3196
Beer	0.1999
Wine	0.3024
Other alcoholic beverages	−0.1544
Sauces	−0.2222
Soups	-
Bouillon	-
Soya products	-
Dietetic products	0.1988
Snacks	−0.3429

## Supplementary Materials

The following are available online at https://www.mdpi.com/2072-6643/12/8/2489/s1, Table S1: Circulating plasma lithium concentration according to season (*n* = 928).
