# Supplementary material for: Plasma Lithium Levels in the General Population: A Cross-Sectional Analysis of Metabolic and Dietary Correlates
Source: Nutrients. 2020 Aug 18;12(8):2489. doi: 10.3390/nu12082489 (PMC7468710; doi:10.3390/nu12082489)
Supplement: Supplementary file 1 [file nutrients-12-02489-s001.pdf]

Supplementay Materials

Plasma Lithium Levels in a General Population: a Cross-Sectional Analysis of Metabolic and Dietary Correlates

by Enderle et al.

Table S1. Circulating plasma lithium concentration according to season (n=928)

	Spring	Sommer	Autumn	Winter	p-value
**Plasma concentration of lithium [µg/L]**	0.90 (0.67; 1.23)	0.98 (0.73; 1.39)	1.01 (0.74; 1.37)	0.98 (0.69; 1.41)	0.18

values are median (Q1; Q2) and p-value is based on Kruskal Wallis test; Abbreviation: Q, quartile
